# Fingerprint patterns in neuropsychological disorder depression among south Indian population

**DOI:** 10.6026/97320630019266

**Published:** 2023-03-31

**Authors:** Hemasankar C, Suresh R Rao, Balaji K, Sai Sushma Yeturu, Ragavendra MP, Madhan Krishnan

**Affiliations:** 1 Department of Physiology, Saveetha Medical College, Saveetha institute of medical and technical sciences, Thandalam, Chennai- 602 105, India; 2 Department of Anatomy, Faculty of Medicine, University of West Indies, Trinidad- 685509, Jamaica; 3 Department of Anatomy, Saveetha Medical College, Saveetha institute of medical and technical sciences, Thandalam, Chennai- 602 105, India; 4 Department of Ophthalmology, Subbaiah Institute of Medical Sciences, Purle, Shivamogga, 577202, Karnataka, India; 5 Department of Pharmacology, Saveetha Medical College, Saveetha institute of medical and technical sciences, Thandalam, Chennai- 602 105, India; 6 Faculty of Allied Health Sciences, Chettinad Hospital and Research Institute, Chettinad Academy of Research and Education, Kelambakkam- 603103, Tamilnadu, India

**Keywords:** Arches, dermatoglyphics, depression, loops, neuropsychological, ridge count, whorls

## Abstract

Depression is a pervasive mental health disorder characterized by persistent sadness and an inability to enjoy activities that were once enjoyable. This study compares the dermatoglyphic patterns of depressed patients with those of healthy, normal individuals in order to determine if dermatoglyphic patterns can be used as biomarkers for early diagnosis and prompt intervention of depression. A total of 100 depressive disorder patients of both sexes between the ages of 18 and 60 were selected for the study. Dermatoglyphic patterns of individual digits were analyzed using the "Ink and Paper" technique. The Whorl, Loop, and Arch patterns, as well as the AFRC, TFRC, and a-b ridge count, were examined using One-Way ANOVA significance and a Chi-Square test using SPSS 20.0. The results showed that the right hand in the depressive diseased group had decreased numbers of Whorls in the 2nd and 5th digits, and decreased numbers of Loops in all five digits. Similarly, decreased numbers of Whorls were found in the 1st, 2nd, 4th, and 5th digits of the left hand, as well as decreased Loops in the 4th digit. However, there were no changes in the Arches of either hand when compared to the control group. The AFRC and TFRC were significantly decreased (p<0.0001), but there was no significant decrease in the a-b ridge count in the depressive diseased group when compared to the control group. Dermatoglyphics, a noninvasive method, can serve as a screening indicator for depressive individuals, the appearance of the decreased count of whorls and loops parameters might be used as an exploratory sign.

## Background:

Depression is one of the most common mental health issues and a leading contributor to disability worldwide. According to the World Health Organization (WHO), in 2015, around 4.4% of the global population was estimated to be suffering from depression. This mental health condition leads to functional impairment, increased medical expenses, and medical symptom burden, making it a serious health care concern. It was also identified as one of the top three causes of disability-adjusted life years due to a study completed by the Global Burden of Disease research in 2010. Depression can cause immense distress and greatly reduce the quality of life [1]. Early diagnosis and treatment of depression can reduce stress on family members and caregivers, as well as medical costs [2]. According to a study by Weyerer et al., elderly people who have been diagnosed with depressive symptoms are found to be more likely to be female, have low education, be divorced, and suffer from concomitant physical illnesses, functional disorders, smoke and drink, and have lower levels of cognitive impairment [3]. In the UAE, depression is a major public health problem, leading to functional impairment, higher medical expenses, and medical symptom burden. Research has also suggested that vitamin D insufficiency may play a significant role in the rise of depression [4]. Studies have shown a correlation between depression and lower levels of vitamin D, further emphasizing the importance of proper mental health care [5, 6]. Mental health issues are a prevalent problem across the country, and awareness of these issues has grown in recent years. It is essential to understand and acknowledge the dangers of these conditions and to provide the necessary care and services to those suffering.

Community samples reveal higher rates of depression in individuals with compared to those without common chronic diseases such as heart disease, hypertension, stroke, diabetes, and chronic lung disease [7, 8]. Cummins first coined the term dermatoglyphics in 1926 to describe this field of science, which has since been accepted across many countries [9]. Dermatoglyphics is a harmonious blend of two words: "derma" meaning skin, and "glyphe" meaning carve, giving the impression that something has been carved out of the skin. It is possible to study the correlation between specific dermatoglyphic characteristics and the underlying pathological processes in many diseases, including psychological disorders [10-11]. This is possible due to the patterns of early differentiation and genetic uniqueness, as well as the relatively easy methods to obtain and store fingerprints. Once fully formed, dermatoglyphic patterns stay unchanged throughout a person's lifetime and are not affected by age or environment. Dermatoglyphics is a useful diagnostic tool for a variety of conditions, including hereditary diseases and mental disorders. There is a wealth of scientific evidence to support this claim, making it a feasible tool to be studied in the context of certain pathological processes [12,13(see PDF), 14]. Mood disorders are complex syndromes that include a range of signs and symptoms sustained over weeks to months and usually recur in a cyclical pattern throughout an individual's lifespan. While unipolar depression is the most commonly encountered mood disorder in clinical practice, the current study attempted to assess the dermatoglyphic patterns in depression disorder patients [15].

Depression is a mental health disorder that affects millions of people around the world, and its impact on the lives of those affected can be devastating. While much is known about its causes and treatments, the role of genetic predisposition in the development of depression has yet to be fully understood. A new study conducted in south India aims to explore the connection between fingerprint patterns and depression, in an effort to gain a better understanding of the genetic basis of this condition. The study will analyze the fingerprint patterns of a group of individuals with depression, and compare them with a control group of people without depression. It is hypothesized that there may be a correlation between certain fingerprint patterns and depression. If such a correlation is found, it could provide important insight into the genetic basis of the disorder. The study will also look at other factors that may be associated with depression, such as lifestyle, family history, social support, and other psychological variables. The results of the study could help to identify people who are more likely to develop depression, as well as individuals who may be more resistant to the disorder. By investigating the role of fingerprint patterns in depression, this study could shed light on the genetic basis of this debilitating mental health disorder. If a correlation between fingerprint patterns and depression is found, it could help to inform both prevention and treatment strategies. The results of this study could help to identify individuals who are at a higher risk of developing depression, and thus, provide them with the information they need to take action to improve their own mental health.

## Methods and Materials:

**Figure 1:**
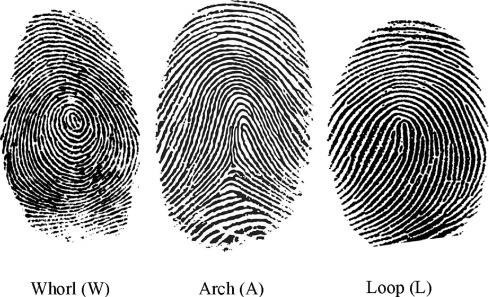
Whorl, Loop, and Arch

**Figure 2:**
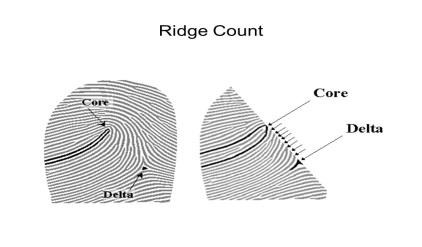
Ridge count

**Figure 3:**
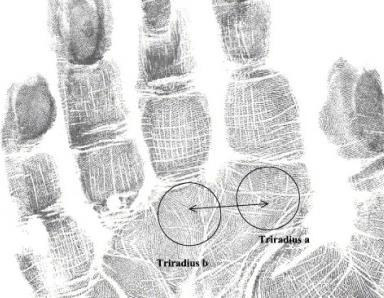
a-b ridge count

In this case-control study, 100 depression disorder patients were recruited from outpatient or inpatient departments of psychiatry at several hospitals in Andhra Pradesh and Karnataka. 100 healthy individuals were chosen as the control group from the area surrounding Sharavathi Dental College in Shivamogga, Karnataka. This study was conducted after obtaining Institutional Human Ethical Committee approval with the reference No: (IRB/IEC, SDC/ET HI/20-21/001). All participants were aged between 18 and 60 years old and had given their informed consent. The findings of each case were recorded in separate forms, and the diagnosis of depressive disorder was based on the criteria laid down in the Diagnostic and Statistical Manual of Mental Disorders (DSM-IV) [16]. Participants with burn scars, infections, or deformities on their fingers or palms were excluded from the study. This research aimed to compare the dermatoglyphic patterns of depression disorder patients to those of healthy individuals, in order to gain a better understanding of the disorder and its associated signs and symptoms. The results of this study may provide valuable insight into the diagnosis and treatment of depression disorder in the future. The participants were identified through a code and their fingerprints were taken using a single-blind method. The palms were scrubbed with water and soap to remove dampness, then cleaned with Ether to remove grease from the surface. The 'ink and paper' technique was used for taking fingerprints, which has been proven to be more effective and convenient than the traditional Cummins ink method [17]. Fingerprints from both hands (all fingers) were taken using the standard method, with the radial edge of the other digits placed downward and away from the body, and the ulnar edge of the thumb placed downward and towards the body. A hand lens and dissecting microscope were used to examine the patterns, which included whorls, loops, arches, and ridges. Whorls are patterns in which the ridges form circuits around a core that can be either circular or elliptical. Whorls have two triradii and come in various shapes, including spiral, double loop, and symmetrical whorls. Symmetrical whorls are composed of concentric ridges that circle a core point, while spiral whorls have a single core and spiral-structured ridges that twine in either a clockwise or anti-clockwise direction. Loops, on the other hand, are simpler compared to whorls. They only have one triradii and their ridges twist at the head of the loop, extending from the opposite extremity of the pattern to the margins of the fingers. If it opens to the ulnar side, it is an ulnar loop, and if it opens to the radial side, it is a radial loop. Arching is a common pattern found in the ridges of the finger that consists of ridges running across the finger and creating a slight distal bow without any triradii. The tented arch is nearly identical to the simple arch, but features a sudden rise of the transversely coursing ridges that give the pattern its name (Figure 1) [18]. In this study, several parameters were measured. Ridge count (RC) is the number of ridges that intersect or touch the line drawn from the easily recognizable triradii (where three ridges meet) to the pattern's core (Figure 2). Total Finger Ridge Count (TFRC) is the sum of the ridge counts of all ten fingers. It represents the pattern's size. Absolute Finger Ridge Count (AFRC) is the total ridge count of all fingers, which reflects the intensity and size of the pattern. The number of ridges between tri radii a and b, located beneath the bases of the index and middle fingers, is referred to as a-b ridge count (Figure 3) [19].

The data was statistically analysed using Chi-square test and One-Way ANOVA, as applicable, with SPSS version 20.0. The results were expressed as mean + SE with P< 0.05 being considered statistically significant.

## Result and Discussion:

The study was conducted with 200 participants (100 with depression and 100 healthy individuals, 50 male and 50 female in each group).

The response rate for the participants was 100%. The results of the fingerprint patterns in the group of patients with depression showed a significantly increased number of whorls in the right hand 1st, 3rd, and 4th digits compared to the control group. However, there was a significant decrease (p<0.0001) in the number of whorls in the right hand 2nd and 5th digits in the depression patients compared to the control group. All five fingerprint digits showed a decrease in the number of loop pattern, although only the 1st and 4thdigits showed a significant (p<0.0001) decrease in loops when compared with the control group. There is a significant increase in number of arches in the right hand 2nd and 5thdigits and significant decrease (p<0.0001) in no of arches in 1st, 3rd and 4th digits in depression patientscompared with control group (Table 1).

**Figure 4:**
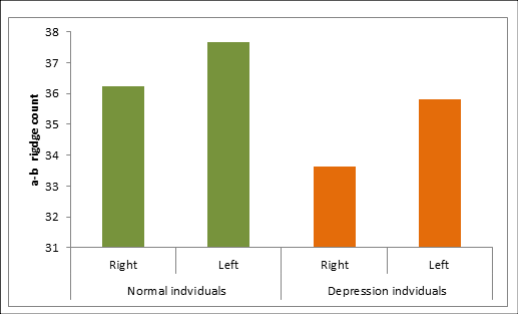
Mean distribution a-b ridge count in normal individual and Depression individuals

**Figure 5:**
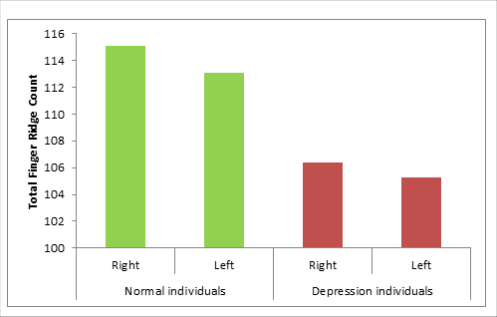
Mean distribution Total finger ridge count (TFRC) in normal individual and Depression individuals

**Figure 6:**
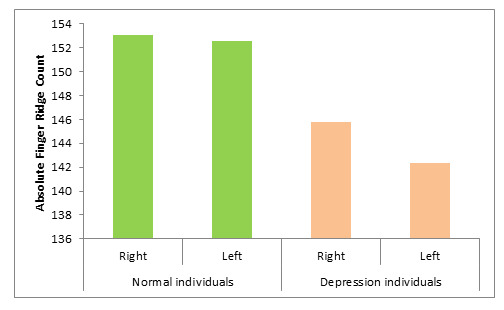
Mean distribution absolute finger ridge count (AFRC) in normal individual and Depression individuals

In our current study, the results of different fingerprint patterns in the depressive disorder patients group showed a significant in the number of whorls in the left hand in digit3 onlywhen compared with the control group and significant decrease (p<0.0001) in number of whorls in the right hand 1st, 2nd, 4th and 5thdigits in depression patients compared with control group. A significant increase in the loop pattern of the fingerprints was found in 2nd,3rd and 5th digits of left hand, but a significant decrease (p<0.0001) in loops was found only in thedigit4 when compared with the control group and no change was found inloop patterns on 1st digit of left hand. There is a significant increase in number of arches in the left hand 1st, 2nd and4th digits and significant decrease (p<0.0001) in number of arches in 3rd and5th digits in depression patients when compared with control group. (Table2) When compared to the control group, the a-b ridge count of depressive disorder patients is highly decreased when compared with the control group, but showed a non-significant (Table 3; Figure 4). When compared to the control group, the depressive diseased group had a significantly decrease in Total finger ridge count (TFRC) (p<0.0001) (Table 4; Figure 5). In depressive diseased group there is a significant decrease in absolute finger ridge count (AFRC) (p<0.0001) compared to the control group (Table 5; Figure 6).

When comparing present study of depression diseases patients with other psychological disorders such as major depressive disorder (MDP), schizophrenia, and bipolar disorder, Yousefi-Nooraie R et al. found that the mean TFRC of MDP was increased compared to the control group [23]. Mahima Shrivastava observed a significant decrease in the TFRC and a-b ridge count in bipolar cases when compared to the control group [24]. Jelovac N (1999) found the palmar ridge counts to be markedly low in bipolar affective disorder compared to controls [25]. Some studies have suggested that total finger ridge counts in patients with schizophrenia are decreased when compared to a normal group, while others have found no significant differences between schizophrenic patients and the control group [26, 27]. At present, there is little published research available that is comparable to the present study on the dermatoglyphics changes of depressive patients. However, the various studies that have been conducted suggest that the changes to the dermatoglyphics pattern of depressive individuals are significant and warrant further investigation. Though some of the parameters in the present study demonstrated an enhancement of dermatoglyphics patterns, the findings were not consistent. Results from this study indicated that there were changes in the dermatoglyphics patterns in depressive disorder patients, making the non-invasive method a potential screening indicator. However, the relevance of these findings needs to be further evaluated with more studies. The study was limited by its small sample size, which may have underreported any significant relationships. Additionally, the discussion of depression-specific patterns for fingerprint analysis was lacking, meaning that further research is needed in order to distinguish between depression and other disorders similar to it, in order to make a prompt diagnosis.

## Conclusion:

In conclusion, the observed changes suggest a significant difference in dermatoglyphics patterns in depressive disorder patients compared to the control group. Dermatoglyphics, as a non-invasive method, can serve as a screening indicator for depressive disorder individuals. The dermatoglyphics variations seen in the study's depressive disorder patients support the hypothesis that palmar patterns are related to the prevalence of depressive illness. Certain dermatoglyphics factors can be used to predict the incidence of depressive disorder to some extent.

## Informed Consent Statement:

Informed consent was obtained from all subjects involved in the study.

## Source of funding:

Self-funding

## Figures and Tables

**Table 1:** Comparison of fingerprint patterns of the right hand in depressive disorder patients and controls (n1=100 & n2=100, P< 0.05*P<0.001**)

**Right Digit 1(Thumb)**
	**Whorl**	**Loop**	**Arch**	**Total**
Control	30	38	32	100
Depression	37	32	31	100
**Chi-square test = 1.262 P = 0.532**
**Right Digit 2 (Index)**
	**Whorl**	**Loop**	**Arch**	**Total**
Control	48	42	10	100
Depression	35	39	26	100
**Chi-square test = 9.258 P = 0.01**
**Right Digit 3 (Middle)**
	**Whorl**	**Loop**	**Arch**	**Total**
Control	40	38	22	100
Depression	47	37	16	100
**Chi-square test = 1.524 P = 0.467**
**Right Digit 4 (Ring)**
	**Whorl**	**Loop**	**Arch**	**Total**
Control	32	43	25	100
Depression	42	37	21	100
**Chi-square test = 2.149 P = 0.341**
**Right Digit 5 (Little)**
	**Whorl**	**Loop**	**Arch**	**Total**
Control	42	36	22	100
Depression	37	33	30	100
**Chi-square test = 1.678 P = 0.432 **

**Table 2:** Comparison of fingerprint patterns of the left hand in depressive disorder patients and controls (n1=100 & n2=100, P< 0.05*P<0.001**)

**Left Digit 1 (Thumb)**
	**Whorl**	**Loop**	**Arch**	**Total**
Control	42	36	22	100
Depression	39	36	25	100
**Chi-square test = 0.303 P = 0.86**
**Left Digit 2 (Index)**
	**Whorl**	**Loop**	**Arch**	**Total**
Control	52	32	16	100
Depression	35	33	32	100
**Chi-square test = 8.671 P = 0.013**
**Left Digit 3 (Middle)**
	**Whorl**	**Loop**	**Arch**	**Total**
Control	40	32	28	100
Depression	48	33	19	100
**Chi-square test = 2.466 P = 0.291**
**Left Digit 4 (Ring)**
	**Whorl**	**Loop**	**Arch**	**Total**
Control	36	46	18	100
Depression	33	38	29	100
**Chi-square test = 3.467 P = 0.177**
**Left Digit 5 (Little)**
Whorl	**Whorl**	**Loop**	**Arch**	**Total**
Control	43	35	22	100
Depression	30	56	14	100
**Chi-square test = 8.939 P = 0.011 **

**Table 3:** Mean distribution a-b ridge count in normal individual and Depression individuals; NS - Not significant

		**Mean**	**SD**	**p-Value**
Right	100	36.23	6.1	
Left	100	37.68	6.51	0.28 (NS)
Right	100	27.37	6.17	
Left	100	29.8	7.01	

**Table 4:** Mean distribution Total finger ridge count (TFRC) in normal individual and Depression individuals

		**Mean**	**SD**	**p-Value**
Right	100	115.4	6.3	
Left	100	113.05	6.15	0.0001
Right	100	106.41	15.96	
Left	100	105.26	15.22	

**Table 5:** Mean distribution absolute finger ridge count (AFRC) in normal individual and Depression individuals

**Number of palms**			**Mean**	**SD**	**p-Value**
Normal individuals	Right	100	153.1	10.99	
	Left	100	152.57	12.91	0.0001
Depression individuals	Right	100	145.82	12.84	
	Left	100	142.32	12.6	
